# Novel optical spectral transmission (OST)-guided versus conventionally disease activity-guided treatment: study protocol of a randomized clinical trial on guidance of a treat-to-target strategy for early rheumatoid arthritis

**DOI:** 10.1186/s13063-019-3285-8

**Published:** 2019-04-17

**Authors:** N. J. Besselink, A. A. A. Westgeest, R. Klaasen, M. Gamala, J. M. van Woerkom, J. Tekstra, M. M. A. Verhoeven, W. E. Van Spil, F. P. J. G. Lafeber, A. C. A. Marijnissen, J. M. Van Laar, J. W. G. Jacobs

**Affiliations:** 1 Rheumatology & Clinical Immunology, University Medical Center Utrecht, G02.230, P.O. Box 85500, 3508GA Utrecht, The Netherlands; 2 Rheumatology, Máxima Medical Center, Eindhoven, The Netherlands; 3 Rheumatology, Meander Medical Center Amersfoort, Amersfoort, The Netherlands; 4 Rheumatology, Noordwest Ziekenhuisgroep, Alkmaar, The Netherlands; 5 Rheumatology, Gelre Ziekenhuizen, Apeldoorn, The Netherlands

**Keywords:** Rheumatoid arthritis, Randomized controlled trial (RCT), Tight-control treatment, Optical spectral transmission (OST) treat-to-target, remission

## Abstract

**Background:**

Assessment of disease activity is a critical component of tight-control, treat-to-target treatment strategies of rheumatoid arthritis (RA). Recently, the HandScan has been validated as a novel method for objectively assessing RA disease activity in only 1.5 min, using optical spectral transmission (OST) in hands and wrists. We describe the protocol of a randomized controlled clinical trial (RCT) to investigate whether HandScan-guided treatment aimed at ‘HandScan remission’ (HandScan arm) is at least as effective as and more cost-effective than clinically guided treatment aimed at ACR/EULAR 2011 Boolean remission (DAS arm).

**Methods/design:**

The study is a multi-center, double-blind, non-inferiority RCT of 18 months duration. Patients ≥ 18 years with newly diagnosed, disease-modifying antirheumatic drug (DMARD)-naïve RA according to the ACR 2010 classification criteria, will be randomized to the DAS arm or the HandScan arm. The efficacy of the arms will be compared by evaluating Health Assessment Questionnaire (HAQ) scores (primary outcome) after 18 months of DMARD therapy, aimed at remission. The equivalence margin in HAQ scores between study arms is 0.2. Secondary outcomes are differences in cost-effectiveness and radiographic joint damage between treatment arms. The non-inferiority sample size calculation to obtain a power of 80% at a one-sided *p* value of 0.05, with 10% dropouts, resulted in 61 patients per arm. In both arms, DMARD strategy will be intensified monthly according to predefined steps until remission is achieved; in both arms DMARDs and treatment steps are identical. If sustained remission, defined as remission that persists consistently over three consecutive months, is achieved, DMARD therapy will be tapered.

**Discussion:**

The study protocol and the specifically designed decision-making software application allow for implementation of this RCT. To test a novel method of assessing disease activity and comparing (cost-)effectiveness with the contemporary method in treat-to-target DMARD strategies in early RA patients.

**Trial registration:**

Dutch Trial Register, NTR6388. Registered on 6 April 2017 (NL50026.041.14). Protocol version 3.0, 19-01-2017.

**Electronic supplementary material:**

The online version of this article (10.1186/s13063-019-3285-8) contains supplementary material, which is available to authorized users.

## Background

Rheumatoid arthritis (RA) is a chronic autoimmune disease with polyarthritis, frequently leading to joint damage and physical disability, especially if not treated adequately as soon as possible after diagnosis. Treatment in the first months is more effective than if the same treatment is applied later in the course of disease [1]. Early and intensive (tight-control) treatment of RA with disease-modifying antirheumatic drugs (DMARDs) has significantly improved RA outcome [2, 3]. The aim of tight-control treatment, i.e., tailoring treatment strategy to the disease activity of individual patients, is to achieve a predefined level of low disease activity, preferably remission (treat-to-target), within a reasonable period of time.

For tight-control and treat-to-target treatment strategies for the treatment of RA, typically frequent disease activity assessment is applied using the Disease Activity Score (DAS), a composite score of an acute phase reactant, such as C-reactive protein (CRP), patient global assessment (PGA), and swollen and tender joint counts (SJC and TJC, respectively) of 28 or 44 joints. Although commonly used in research and daily clinical practice, assessment of the DAS is rather time-consuming and subjective, and the DAS is only validated on the group level [4]. However, fast, objective, and easily implementable tools to assess arthritis and disease activity are lacking [5]. The HandScan (Hemics BV), using optical spectral transmission (OST), objectively measures the reduced transmission of light through joint tissues in the presence of inflammation (e.g., synovitis, tenosynovitis) [6, 7]. HandScan results reflect disease activity, correlating moderately with DAS28 (r = 0.42, *p* = 0.001) [6]. Moreover, HandScan correlates moderately with ultrasound (US) assessing synovial inflammation of hand and wrist joints (Spearman’s correlation coefficient, ρ = 0.54, 95% CI 0.28 to 0.73, *p* < 0.01), while DAS28 did not correlate with these US results (ρ = 0.06, 95% CI − 0.26 to 0.36, *p* = 0.71) [7]. Test-retest reliability of the HandScan was excellent at both the patient level (ICC = 0.86, 95% CI 0.76 to 0.92, *p* < 0.001) and joint level (ICC = 0.76, 95% CI 0.73 to 0.79, *p* < 0.001) [7]. In addition, HandScan has proven to be user-friendly—i.e., an assistant without medical background can operate the device—and fast (it provides the inflammation score within 1.5 min).

The aim of this study protocol, designed according to the SPIRIT 2013 Checklist (Additional file 1), was to determine the applicability of the HandScan in tight-control and treat-to-target treatment strategies of early RA patients. To facilitate this, a specifically designed decision-making software application was developed, allowing for double-blind comparison of HandScan-guided treatment with the contemporary method of DAS-guided treatment.

## Methods/design

This is an investigator-initiated, multi-center, double-blind non-inferiority randomized controlled trial (RCT) of 18 months duration. Patients will be randomized (1:1) to a HandScan-guided treatment aimed at HandScan remission (HandScan arm) or a clinically DAS-guided treatment aimed at ACR/EULAR 2011 Boolean remission (DAS arm). The study is performed at six departments of rheumatology in the Netherlands, the University Medical Center Utrecht (UMCU), and five non-university hospitals: Meander Medical Center Amersfoort, Noord West Ziekenhuizen Alkmaar, Máxima Medisch Centrum Eindhoven, and Gelre Ziekenhuizen Apeldoorn. The study has been approved by the Ethical Committee of the University Medical Center Utrecht, Utrecht, The Netherlands on April 6, 2017 (NL50026.041.14), and has been registered in the Dutch Trial Register (NTR6388). Privacy of patients will be protected according to the General Data Protection Regulation, using anonymized data.

### Objectives and primary and secondary outcomes and their rationale

The overall aim of the study described in this protocol is to demonstrate clinical efficacy and cost-effectiveness of the HandScan arm compared to the DAS arm, with identical treatment and treatment steps in both arms. The primary outcome is the change in Health Assessment Questionnaire (HAQ) score from baseline to 18 months, reflecting both actual disease activity and physical disability as a measure for cumulative past disease activity [8]. We chose not to use DAS remission or the Boolean remission criteria with the components of DAS as primary outcome, first, because this would probably favor the DAS arm of our trial over the HandScan arm and, second, because DAS-based remission criteria have a relatively high risk of false negative classification of remission. This is caused by high scores on PGA and TJC in case of concomitant soft tissue rheumatism, fibromyalgia, or other non-inflammatory pain of chronic pain syndrome [9].

Secondary outcomes are the change in HAQ score over time, cost-effectiveness, based on customized cost questionnaires (including direct and indirect costs), and radiographic damage of hand and wrist joints, assessed using a newly developed fully automated radiographic scoring system of joint width of hand and wrist joints [10], as well as the conventional Sharp van der Heijde (SvdH) score of joint width and erosions of hands, wrists, and feet [11].

### Patients

Patients ≥ 18 years with newly diagnosed DMARD-naïve RA according to the ACR/EULAR 2010 classification criteria [12] will be eligible for this study. Detailed selection criteria are shown in Table 1.

**Table 1:** Selection criteria

Inclusion criteria	
• Early (< 1 year) RA, fulfilling 2010 ACR/EULAR criteria	
• Age ≥ 18 years	
• Ability and willingness to give written informed consent	
• Ability to comply with the study protocol	
Exclusion criteria	
• Significant visual deformations of hands or fingers (impeding HandScan analysis)	
*Other (joint) disease*	
• Concomitant or current inflammatory joint disease other than RA	
• Porphyria (HandScan risk analysis).	
*Drug-specific*	
• Contraindication for methotrexate or prednisolone	
• Glucocorticoids used for RA < 6 weeks prior to baseline (NB, inhaled glucocorticoids are allowed)	
• Previous treatment with any (biological) DMARD that is used in the treatment of RA	
• Treatment with any investigational agent within 4 weeks or period of five half-lives, whichever is longer, before screening	
• Patients using photodynamic therapy medication (HandScan risk analysis)	
*General medical*	
• Pregnancy or breast-feeding	
• History of alcohol or substance abuse within the 6 months prior to screening. Alcohol abuse is defined as more than 3 units per day	
• Neuropathies or other painful conditions that might interfere with pain evaluation	

All participating patients provide written informed consent, according to the ethical principles from the Declaration of Helsinki. At the screening visit, informed consent is signed by the patient and research/rheumatology nurse or investigator, and selection criteria are checked.

### Randomization

Randomization lists per center will be prepared at UMC Utrecht, using *nQuery Advisor* in permuted blocks of 4; randomization lists are safeguarded at the UMC Utrecht. Randomization data will be incorporated in the decision-making software application that is used in each of the institutes. This way, on-site research staff, patients, and sponsor trial personnel will remain blinded to treatment strategy. A patient will be allocated to the HandScan or DAS arm, as soon as baseline clinical data are entered into the software application. A patient-specific study number and the year of birth will function as identifiers. Unblinding will not be necessary as both arms receive identical medication in an open fashion.

### Assessments

**Fig. 1:**
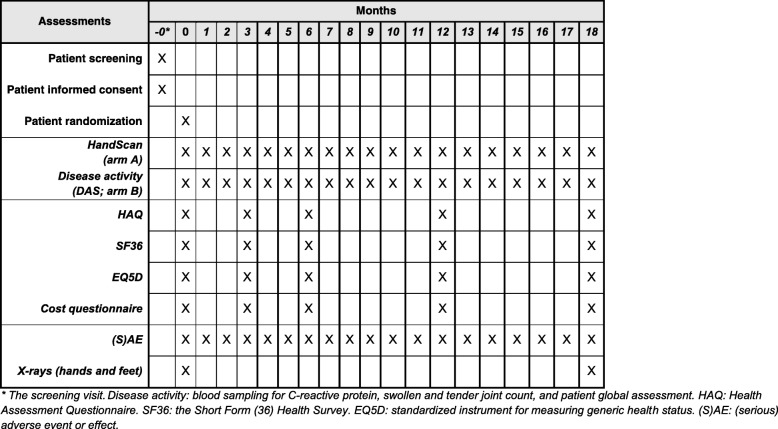
SPIRIT figure, trial visits, and assessments

Patients will visit the outpatient clinics monthly (Fig. 1). At each visit, disease activity will be measured, to ensure blinding both with HandScan and DAS44, consisting of serum CRP, a 44 joints assessment for swelling and tenderness, and a PGA (VAS 0–10 cm, 10 = worst). At baseline and 3, 6, 12, and 18 months patients will fill out the HAQ (first outcome), the Short Form Health Survey (SF36), EuroQol (EQ5D), and the questionnaire on direct and indirect costs (second outcome). Direct costs (health care) and indirect costs (loss of paid and household productivity) will be calculated from questionnaires, including the Health and Labour questionnaire [13]. Radiographs in the antero-posterior direction of hands, wrists, and feet will be obtained at baseline and 18 months (second outcome). The current gold standard for scoring these radiographs is the Sharp/van der Heijde scoring method [11]. This method scores erosions (score 0–5 per region) and joint space narrowing (score 0–4 per region) of these joints and sums the scores to the SHS score, ranging from 0 to 448. A newly developed fully automated radiographic scoring system of hand and wrist joints is also applied to quantify changes in the joint space width over time.

At each visit, patients’ blood will be sampled (10 cc) for CRP evaluation and to monitor for adverse-effects of medication. Data from all centers are collected digitally in one eCRF, through the online data-gathering tool *Research Online* (developed by UMC Utrecht Julius Center).

### Definitions of remission

Aimed at predefined remission criteria, treatment is intensified in a similar fashion for both arms, according to a predefined schedule. In the DAS arm, remission is defined based on the ACR/EULAR 2011 Boolean remission criteria, all of which must be met [14]:

- TJC ≤ 1 of 44 joints: sternoclavicular, acromioclavicular, shoulder, elbow, wrist, metacarpophalangeal (MCP), proximal interphalangeal (PIP), metatarsophalangeal (MTP), knee, and ankle joints
- SJC of 30 small joints (MCP, PIP, and MTP joints) ≤ 1 AND SJC of 14 large and other joints (sternoclavicular, acromioclavicular, shoulder, elbow, wrist, knee, and ankle joints) = 0
- CRP ≤ 1 mg/dl
- PGA ≤ 1 (on VAS 0–10 cm, 10 = worst)

In the HandScan arm, remission is defined based on the following HandScan criteria, all of which must be met:

- Total optical joint score per patient ≤ 11(based on ROC curves in a comparative study with DAS28 and ultrasonography) [6, 7]
- ≤ 1 joint per patient with an optical score of > 1

**Fig. 2:**
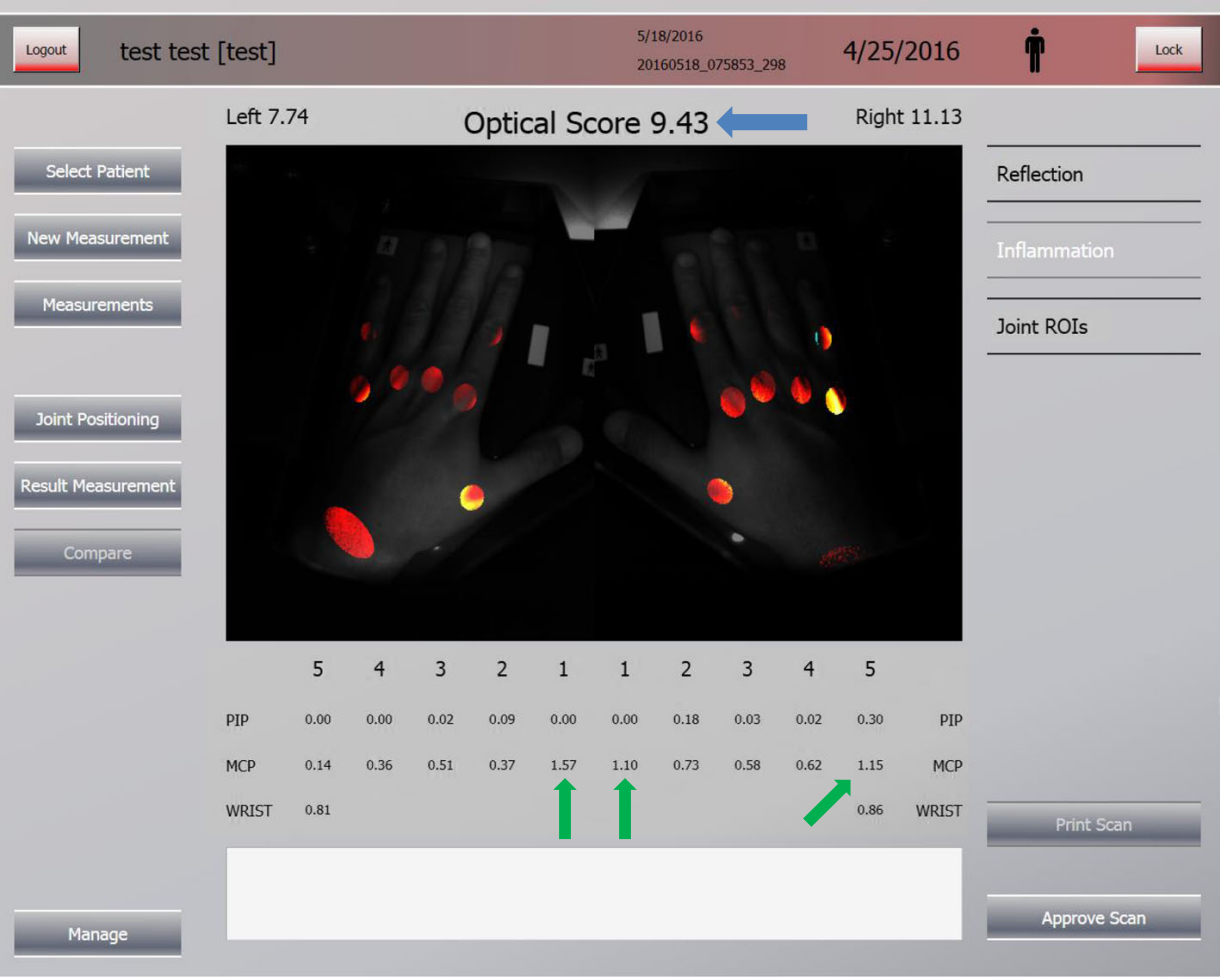
The HandScan user interface; total and individual optical joint scores. The total optical joint score of 9.43 (*blue arrow*) meets the HandScan remission criterion for total score. Individual optical joint criteria are shown below the picture. Three joints (*green arrows*) exceed the individual optical joint score criterion (> 1). Considering the HandScan remission criteria (total optical spectral transmission (OST) score ≤ 11 AND a maximum of one joint with OST > 1), this (test) patient is not in remission

The optical joint scores are shown by the HandScan shortly after assessment (see screen shot of the HandScan in Fig. 2).

### Treatment strategy

After randomization, in both arms all patients will initiate a methotrexate (MTX)-based tight-control strategy, with 10 mg per week MTX orally as starting dose and with prednisolone (PRED) fixed dose of 10 mg/day orally [15]. Patients are evaluated monthly and at each visit a dosage decision is made based on efficacy and adverse events. In case remission is not achieved, MTX dose will be increased at each monthly visit until either remission or the maximum tolerable dose (MTD) is reached (Table 2). Escalation steps are 15, 20, 25, 30 mg MTX/week orally, followed by 30 mg MTX/wk (or MTD) subcutaneously. At a dose of MTX of 25 mg/week (or MTD of MTX), hydroxychloroquine (HCQ) 400 mg/day for patients ≥ 60 kg (or 200 mg/day for patients ≤ 60 kg) will be added. After the final escalation step of MTX, in case of no remission, tumor necrosis factor inhibitor (anti-TNF), e.g., adalimumab 40 mg s.c. every 2 weeks, will be added to the MTX therapy, while HCQ will be stopped.

**Table 2:** Intensifying treatment strategy in case remission is not achieved

Week	MTX*	PRED	HCQ	Anti-TNF
	mg/week	10 mg/day	400 mg/day	
0 = start of study	10	+	–	–
+ 4	15	+	–	–
+ 4	20	+	–	–
+ 4	25	+	+	–
+ 4	30	+	+	–
+ 4	Same dose s.c.	+	+	–
+ 4	Same dose s.c.	+	–	+
+ 4	Same dose s.c.	+	–	+
+ 4	Same dose s.c.	+	–	+

If remission is not achieved after each period of 4 weeks, treatment is intensified stepwise. *MTX* methotrexate, *PRED* prednisolone, *HCQ* hydroxychloroquine, *anti-TNF* anti-tumor necrosis factor, *s.c.* subcutaneously

*Same dose s.c.: the dose at the previous step, given s.c. at a dose of 30 mg, or earlier. In case of dose-dependent adverse reactions to MTX (> 10 mg/week), previously tolerated dose will be administered s.c., and this will then be the maximum tolerable dose (MTD) for that patient. Further intensifying treatment would be adding or continuing HCQ. HCQ is given for three consecutive months in every scenario

**Fig. 3:**
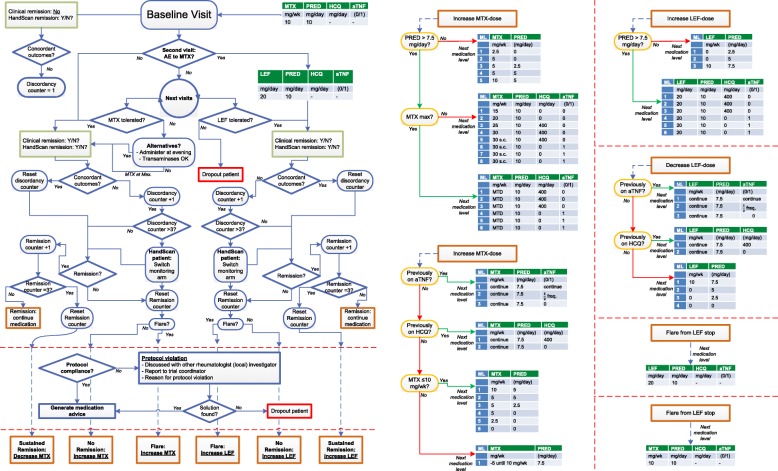
Flowchart. *Green boxes* indicate assessment of remission. *Brown boxes* indicate medication dosage-related events (maintaining, intensifying, and tapering treatment). *Red boxes* indicate patient dropouts. *Blue diamonds* indicate decisions for patient-tailored treatment. *Orange boxes* refer to current medication dosages. *Clinical remission* is remission according to ACR/EULAR 2011 Boolean remission criteria [10]. *HandScan remission* remission is achieved if total optical spectral transmission (OST) score ≤ 11 AND a maximum of one joint with OST > 1. *MTX* methotrexate, *LEF* leflunomide, *PRED* prednisolone, *HCQ* hydroxychloroquine, *aTNF* tumour necrosis factor inhibitor, *MTX at max* maximum (tolerable) dose, i.e., 30 mg or lower, or maximal tolerable dose, *AE* adverse event or effect, *ML* medication level

If remission is achieved, treatment will be continued unchanged. If remission persists over three consecutive months (sustained remission (SR)), treatment intensity will be de-escalated by one step; following de-escalation steps will be taken every time remission persists for another 3 months (Table 3). De-escalation steps will vary with the dosages at remission (Fig. 3 and Table 3), but the first de-escalation step will always be tapering PRED to 7.5 mg/day. If disease activity flares (i.e., loss of remission according to the remission criteria), patients will return to the previous dosages at which they achieved remission and medication will be escalated until remission is achieved again. If disease activity flares during medication-free remission, patients will return to MTX 10 mg/week and PRED 10 mg/day (medication at start of the study) and medication will be escalated until remission is achieved again. The treatment protocol is derived from the second Computer Assisted Management in Early Rheumatoid Arthritis trial (CAMERA-II) [15]. Co-medications are calcium and vitamin D supplementation and a bisphosphonate during PRED treatment, and folic acid to prevent MTX toxicity; dosages of co-medication are according to guidelines. Each center will provide their own preferred anti-TNF.

**Table 3:** Tapering and stopping treatment in case remission is maintained after 12 weeks of unchanged treatment*

	Weeks of remission	MTX	PRED	HCQ	Anti-TNF
		(mg/week)	(mg/day)	(mg/day)	
MTX + PRED	< 12	Same dose	10	–	–
	12	Same dose	7.5	–	–
	> 12, every 12 weeks	Decrease with 5 mg/week until 10 mg/week	7.5	–	–
	+ 12	10	5	–	–
	+ 12	5	5	–	–
	+ 12	5	2.5	–	–
	+ 12	5	Stop	–	–
	+ 12	2.5	–	–	–
	+ 12	Stop	–	–	–
MTX + PRED + HCQ	< 12	Same dose	10	400	–
	12	Same dose	7.5	400	–
	> 12, every 12 weeks	Decrease with 5 mg/week until 10 mg/week	7.5	stop	–
	+ 12	10	5	–	–
	+ 12	5	5	–	–
	+ 12	5	2.5	–	–
	+ 12	5	Stop	–	–
	+ 12	2.5	–	–	–
	+ 12	Stop	–	–	–
MTX + PRED + aTNF	< 12	Same dose	10	–	Same dose
	12	Same dose	7.5	–	Same dose
	> 12,	Same dose	7.5	–	½ frequency^#^
	every 12 weeks	One step back until 10 mg/week	7.5	–	Stop
	+ 12	10	5	–	–
	+ 12	5	5	–	–
	+ 12	5	2.5	–	–
	+ 12	5	Stop	–	–
	+ 12	2.5	–	–	–
	+ 12	Stop	–	–	–

* Tapering treatment depends on the combination of medication at the moment of sustained remission. ^#^ In this multi-center study, centers prescribe their preferential anti-TNF; therefore, a more global approach to decreasing aTNF dose is applied—reduction of frequency of administration, i.e., extension of dosing interval

*MTX* methotrexate, *PRED* prednisone, *HCQ* hydroxychloroquine, *anti/a-TNF* anti-tumor necrosis factor

### Adverse reactions to DMARDs

In case of adverse reactions to initial dose of MTX (10 mg/week), MTX will be substituted with leflunomide (LEF) 20 mg/day. If LEF is well tolerated and remission is not achieved, HCQ will be added; next escalation steps are as described above (Table 2). Patients that switch to LEF and are consequently unable to achieve remission will reach anti-TNF treatment faster than patients on MTX (with the exception of an MTD). In case of adverse reactions to MTX at dosages ≥ 10 mg/week, the last well-tolerated dose will be administered subcutaneously and considered to be the MTD. If remission is not achieved, treatment will be intensified according to Table 2, e.g., at a MTD of MTX 15 mg/week subcutaneously, HCQ will be added.

### Implementation of a software application for patient-tailored, tight-control treatment

Although treatment regimens and dosing steps are identical for all patients, actual medication will differ considerably between patients in this study, as the study protocol tailors treatment to every patient in a treat-to-target strategy (Fig. 3). To account for these variations in treatments and to allow for double-blind treatment, a decision-making software application was developed. It uses a data trail (data log) of current and previous patient-specific input to check, e.g., for consistent discordance between remission criteria and prevent drug dosages higher than previous MTD in case of a flare. At each visit, the patient identification number, year of birth, and parameters for both clinically and HandScan-assessed disease activity will be entered into the application. Based on whether there is remission or not, according to the criteria of the randomized arm, the application will provide the next patient-specific medication step.

The application was developed and tested to conform to the regulations and guidelines for the development of a medical appliance class I and medical software class B (*EN 62304 Medical device software –Software life-cycle processes, EN 62366 Usability, ISO 14971 Medical devices – Application of risk management to medical devices*).

### Prevention of under- and overtreatment in the HandScan arm

Since treatment guidance by the HandScan is a novel approach, measures are taken to prevent large deviation of the HandScan-guided strategy from the DAS guided strategy to avoid under- or overtreatment. If treatment decisions dictated by HandScan at three consecutive monthly visits (Fig. 3) are discordant with decisions that would have been dictated by DAS, patients will be switched to the DAS-guided arm and medication decisions will be immediately guided by this arm. Discordancies are tracked in the software application; the switch is implemented without intervention of the researcher, blinded for researcher and patient. This way, patients in the HandScan arm might potentially be under- or overtreated (based on DAS guidance) only for a maximum of two consecutive months.

### Statistical analyses

#### Sample size

Sample size was calculated for the primary outcome, HAQ score, and the primary analysis of the non-inferiority design using two-sample *t*-tests and data from the CAMERA-II trial: mean (SD) HAQ score at 18 months 0.38(0.4) [15]. An equivalence margin of 0.2 in HAQ scores between study arms was considered clinically acceptable. A sample size of 51 per group was calculated to obtain a power of 80% at a one-sided *p* value of 0.05. Taking into account 10% drop-out, total sample size was set at 112 patients.

#### Primary outcome analyses

The primary outcome is HAQ score; the primary analysis is a general linear model, testing HAQ scores at 18 months between the two arms according to intention to treat (ITT), controlling for baseline HAQ, center (stratification factor in the randomization), and the baseline covariates age, gender, disease activity (DAS28), rheumatoid factor, and anti-CCP. The ITT population will include all randomized patients as long as they have taken study medication at least once and at least one efficacy measurement was obtained. Secondary analyses are 1) the primary analysis, but for the per protocol population (i.e., all patients without major protocol violations or a switch between study arms), and 2) a mixed model analysis comparing HAQ scores over time (baseline and 3, 6, 12, and 18 months) between the study arms. In this model we will control for the same covariates as those in the primary analysis. Although this model handles missing data well, if ≥ 10% of data are missing, as a sensitivity analysis data will be analyzed after multiple imputation if data seem to be missing at random.

#### Secondary outcomes

Quality of life will be evaluated at baseline and after 3, 6, 12, and 18 months using SF36 and EQ5D questionnaires with mixed model analyses between study arms.

Cost-effectiveness of the HandScan arm versus the DAS arm treatment will be calculated from actual data (i.e., a trial-based economic evaluation). In the HandScan arm, cost for a rheumatologist at the clinical visits will only be included if a rheumatologist would have actually been required, e.g., to change medication. To prevent overestimation of cost of rheumatologist time, a visit rate of once per month during the first 6 months and once per 3 months thereafter will be assumed for cost calculation. Cost-effectiveness planes and acceptability curves will also be estimated from the societal (base case), healthcare, and hospital perspectives. Differences in quality-adjusted life years (QALYs; life years multiplied by the utility value, as calculated using EQ5D) and costs (for drug cost, other direct costs, and indirect costs) will be calculated using bootstrapping (5000 resamplings, with replacement). Costs and QALYs will be discounted by 4% and 1.5% according to the Dutch guidelines for pharmaco-economic evaluations. Sensitivity analyses will be performed for time spent by rheumatologists, number of visits, QALYs (according to either EQ5D or SF36), costing method (Human Capital Approach or Friction costs method), and discount rates. Missing data for costs and QALY calculation will be imputed using multiple imputation.

Radiographic joint space width and bone erosions of hands and feet will be measured by SvdH score, [16] total score, and separately for joint space narrowing and erosion scores. Differences in change at 18 months from baseline between arms will be tested for statistically significant differences with Mann-Whitney U tests, and with generalized linear mixed models. As sensitivity analysis, joint space narrowing scores by a novel [10], fully automated assessment will be performed and analyzed. Moreover, cumulative disease activity according to area under the curve of HandScan data in each arm will be correlated to radiographic damage (total, narrowing, and erosion scores) by SvdH and automated assessments by Spearman correlation analyses.

### Patient safety

An independent researcher will analyze the number of study arm switches when the first 20 patients have 6 months of follow-up data. If more than ten patients need to switch to the DAS-guided arm because of discordant treatment decisions for three consecutive months, inclusion will be stopped. The patients who have been included up to that moment will continue to be followed-up, to obtain sufficient information for the evaluation of the HandScan functionality in tight-control treatment strategy. If more than 20% of the first 20 patients have protocol violations based on clinical judgment within 6 months after inclusion, the medical research ethics committee (MREC) will be informed and protocol modifications deemed necessary will be communicated with the site investigators, treating rheumatologists, and, if necessary, trial participants. All adverse events reported by trial participants or observed by investigator staff will be recorded. As the study does not involve experimental medication or treatment, no safety analysis will be performed and an independent data safety and monitoring board will not be installed.

## Discussion

This study aims to evaluate clinical efficacy of a HandScan-guided versus DAS-guided tight-control and treat-to-target treatment strategy for early RA. The HandScan guidance has potential drawbacks, mainly because it relies solely on arthritic activity in hands and wrists. However, optical spectral transmission (OST) did correlate to DAS28 in a previous study [6], as well as to US assessed synovial inflammation of hand and wrist joints, while DAS28 did not correlate with these US results. It is possible that, considering the strengths and weaknesses of both guidance methods, the optimal guidance for future treatment of early RA would be using both methods or a combination of parameters of both methods (e.g., HandScan with CRP measurement).

An economic evaluation of guidance of a tight-control strategy using the HandScan has been previously published [17]. Implementation of the HandScan as a monitoring tool was modeled at comparable costs and comparable effects as using clinical assessments. To validate this result, cost-effectiveness in the current study will be calculated based on actual data (i.e., a trial-based economic evaluation); this approach requires less assumptions and therefore has a lower risk of bias.

This protocol describes a specifically designed software application to allow for double-blind, safe, and patient-tailored guidance of treatment. Implementation of the software application has the advantage that it allows for on-site patient randomization and double-blind (for the guidance method) treatment. Deviation from standard treatment schedules is allowed, for example, by defining a MTD or the opportunity to switch from MTX to LEF in case of MTX intolerance. Three consecutive discrepancies in medication advice between the HandScan and DAS arm lead to switch of the respective patients in the HandScan arm to the DAS arm as a built-in safety for potentially large differences between arms. Importantly, as neither the patient nor the physician is aware of a switch, the double-blind design will be maintained.

This specifically designed decision-making software application also allows for implementation of other RCTs testing future, novel methods of guidance of tight-control and treat-to-target treatment strategies in RA.

## Trial status

The trial started April 4, 2017 and is currently recruiting.

## Additional file

Additional file 1: SPIRIT 2013 Checklist: Recommended items to address in a clinical trial protocol and related documents*. (DOC 121 kb)
